# State-of-the-art imaging in oesophago-gastric cancer

**DOI:** 10.1259/bjr.20220410

**Published:** 2022-06-15

**Authors:** Samuel J Withey, Vicky Goh, Kieran G Foley

**Affiliations:** Department of Radiology, The Royal Marsden NHS Foundation Trust, London, UK; Cancer Imaging, School of Biomedical Engineering & Imaging Sciences, King’s College London, London, UK; Department of Radiology, Guy’s and St Thomas’ NHS Foundation Trust, London, UK; Division of Cancer & Genetics, School of Medicine, Cardiff University, Wales, UK; Department of Radiology, Velindre Cancer Centre, Cardiff, UK

## Abstract

Radiological investigations are essential in the management of oesophageal and gastro-oesophageal junction cancers. The current multimodal combination of CT, 18F-fluorodeoxyglucose positron emission tomography combined with CT (PET/CT) and endoscopic ultrasound (EUS) has limitations, which hinders the prognostic and predictive information that can be used to guide optimum treatment decisions. Therefore, the development of improved imaging techniques is vital to improve patient management. This review describes the current evidence for state-of-the-art imaging techniques in oesophago-gastric cancer including high resolution MRI, diffusion-weighted MRI, dynamic contrast-enhanced MRI, whole-body MRI, perfusion CT, novel PET tracers, and integrated PET/MRI. These novel imaging techniques may help clinicians improve the diagnosis, staging, treatment planning, and response assessment of oesophago-gastric cancer.

## Introduction

Around 9000 cases of oesophageal and gastro-oesophageal junction (GOJ) cancers (referred to collectively in this review as oesophago-gastric cancers) are diagnosed in the UK each year and the incidence has risen by 6% over the past 20 years.^1^ Worldwide, there are more than 600,000 new cases each year.^2^ Squamous cell carcinoma (SCC) is the most common subtype worldwide but adenocarcinoma is more common in the UK, USA, and Western Europe.^3^ Both are associated with poor prognosis, with 5-year survival reported at 12% for SCC and 15% for adenocarcinoma.^4^

SCCs are distributed equally between the upper and mid-thoracic oesophagus, whereas most adenocarcinomas are located in the distal oesophagus or at the GOJ.^5^ Using the current TNM eighth edition classification, Siewert Type 1 and 2 GOJ tumours are staged as oesophageal cancers, and Siewert Type 3 tumours (with the epicentre in the proximal stomach between 2 and 5 cm from the GOJ) are staged as gastric cancers.^6^ The oesophagus has a rich bidirectional lymphatic drainage system, meaning lymph node metastases (and satellite nodules) can develop along the entire length of the oesophagus.^7^ Histological subtype is generally not taken into account when assigning the TNM classification, but the overall stage groups have subtle differences between subtypes.^6^

In terms of management, tumours confined to the mucosa (T1a) can be considered for endoscopic resection or ablation, whereas tumours involving the submucosa (T1b) usually require oesophagectomy due to higher rates of occult lymph node metastases.^8^ Neoadjuvant therapy provides a survival benefit for patients with locally advanced disease but the benefit reduces in early tumours.^9^ Regional nodal involvement is a further indication for neoadjuvant therapy.^9^ Distant metastatic disease is present in up to 50% of patients at diagnosis, and generally precludes surgical management,^10^ although the value of surgical and ablative techniques in oligometastatic disease is being explored.^11^

Imaging is essential for all aspects of oesophago-gastric cancer management. In this review, we summarise current best practice and highlight how state-of-the-art imaging can improve diagnosis and staging, allow more effective treatment planning and monitoring, and improve risk stratification.

### Current best practice in diagnosis and staging

**Figure 1.**
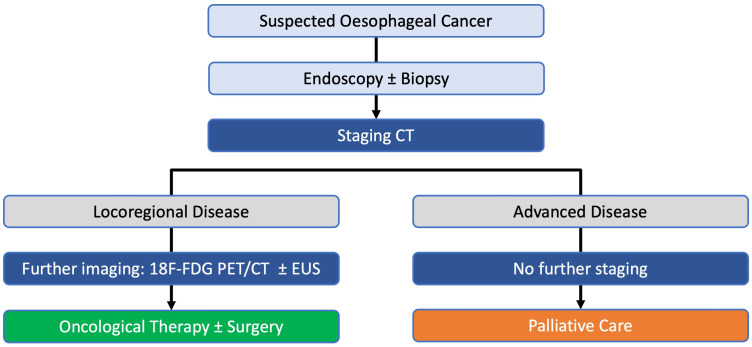
Typical radiological staging pathway for most patients diagnosed with oesophago-gastric cancer in the United Kingdom. EUS, endoscopic ultrasound; PET, positron emission tomography.

Oesophago-gastric cancer is usually diagnosed following first-line endoscopy and biopsy, but disease staging is largely influenced by radiological techniques. A patient staging algorithm describing the current radiological pathway is in shown in Figure 1. The TNM v. 8.0 staging classification for oesophago-gastric cancer is described in Table 1.

**Table 1.** TNM staging system for oesophageal carcinomas

T Category	T Criteria
TX	Tumour cannot be assessed
T0	No evidence of primary tumour
Tis	High-grade dysplasia, defined as malignant cells confined to the epithelium by the basement membrane
T1a	Tumour invades the lamina propria or muscularis mucosae
T1b	Tumour invades the submucosa
T2	Tumour invades the muscularis propria
T3	Tumour invades adventitia
T4a	Tumour invades the pleura, pericardium, azygos vein, diaphragm, or peritoneum
T4b	Tumour invades other adjacent structures, such as the aorta, vertebral body, or airway
**N Category**	**N Criteria**
NX	Regional lymph nodes cannot be assessed
N0	No regional lymph node metastasis
N1	Metastases in one or two regional lymph nodes
N2	Metastases in three to six regional lymph nodes
N3	Metastases in seven or more regional lymph nodes
**M Category**	**M Criteria**
M0	No distant metastases
M1	Distant metastases

Reproduced from: AJCC Cancer Staging Manual. eighth Edition ed: Springer International Publishing, 2017.

### CT

**Figure 2.**
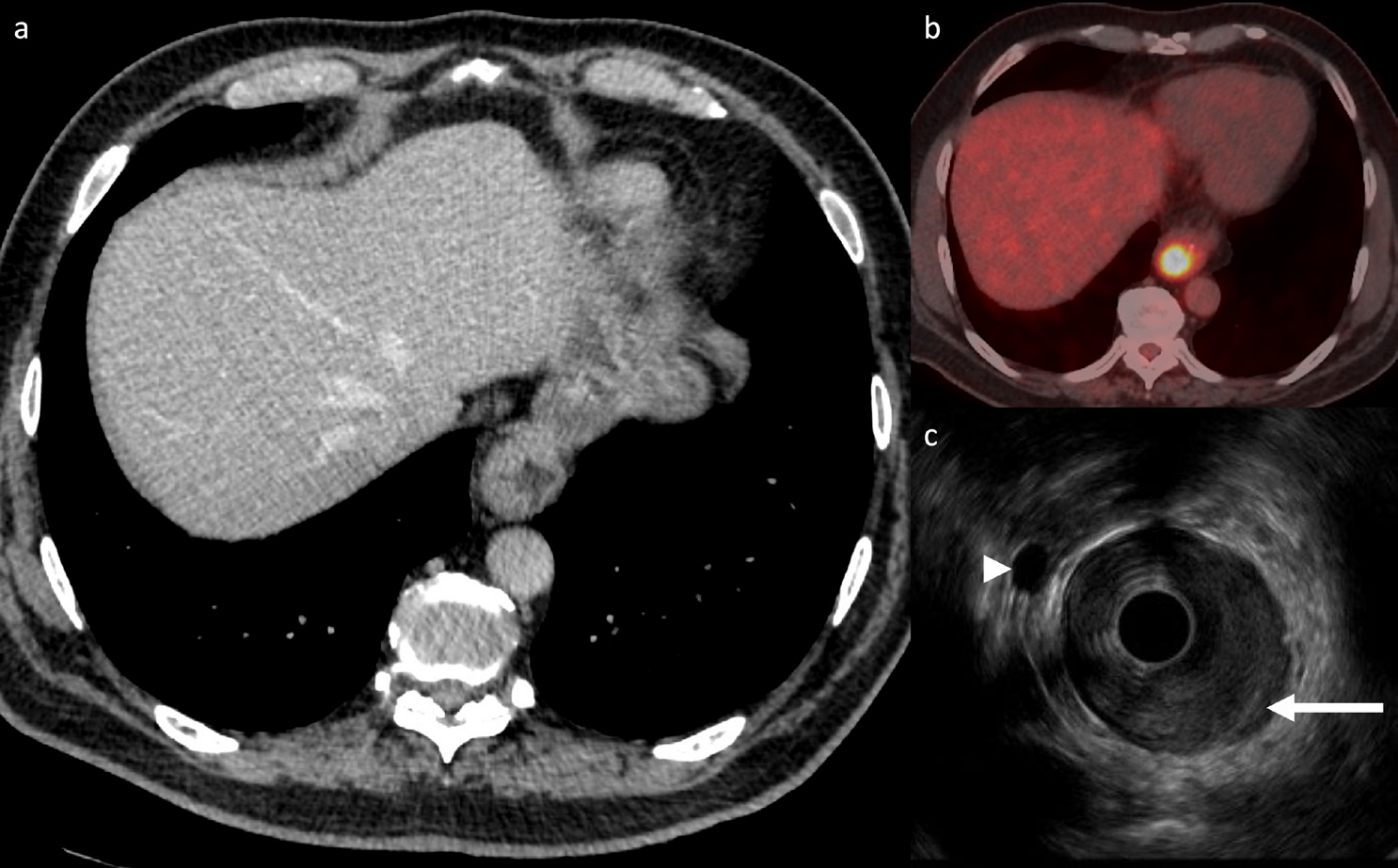
Axial CT showing mural thickening of the distal oesophagus (a), axial fused ^18^F-FDG PET/CT (b) showing FDG uptake in the tumour. Clinical staging of both CT and PET/CT was cT3 N0 M0. Endoscopic ultrasound (c) showed that the distal oesophageal tumour (arrow) involved the diaphragmatic crus distally and there was a malignant perioesophageal lymph node (arrowhead), therefore the final staging was cT4a N1 M0 (Images courtesy of Dr K G Foley, Velindre Cancer Centre). FDG, fluorodeoxyglucose; PET, positron emission tomography.

Contrast-enhanced CT is the primary radiological staging investigation (Figure 2), usually performed after endoscopy and biopsy.^12,13^ CT can identify patients with clear distant metastatic disease which usually signals palliative therapy and precludes futile radical treatment. However, CT cannot identify the layers of the oesophageal wall, therefore is inaccurate for early T-staging.^14^ When dichotimising early (T1-T2) *vs* late (T3-T4) oesophageal cancer, CT has a reported diagnostic accuracy of 80–82% (*n* = 74 patients).^15^ Using a 1 cm short-axis size threshold, the sensitivity in diagnosing lymph node metastases has been reported as 50%, and specificity as 83%.^16^

### Endoscopic ultrasound

Endoscopic ultrasound (EUS) is used in some centres for more detailed locoregional staging (Figure 2).^16^ EUS may be used to differentiate between T1a and T1b tumours^17^ although when dichotimising T1-T2 *vs* T3-T4 oesophageal cancer, EUS has a reported diagnostic accuracy of 81–85% (*n* = 74 patients).^15^ Its sensitivity and specificity for nodal metastases is 80 and 70%, respectively.^16^ A benefit of EUS is that it provides an opportunity to perform fine needle aspiration (FNA) of suspicious nodes, increasing its diagnostic accuracy from 74 to 87%.^18^ The limitations of EUS include its availability, accuracy related to operator experience, and stenotic tumours not traversable by the endoscope.^19^

### PET/CT

18-Fluorine fluorodeoxyglucose (^18^F-FDG) PET/CT is recommended for assessment of metastatic disease undetected by CT in those planned for curative treatment. ^18^F-FDG PET/CT has a greater sensitivity for distant metastases than CT (71% *vs*  52%).^16^ However, the sensitivity for perioesophageal nodal disease is poor, reported at 57%, with 85% specificity.^16^ This is, in part, due to the spatial resolution of PET limiting the differentiation of perioesophageal nodes from the primary tumour. Use of ^18^F-FDG PET/CT has been reported to improve patient stratification, reduce relapse rate, and increase overall survival after oesophagectomy.^20^ In surgical candidates, clinically relevant changes to staging have been reported in around 24%, mainly related to upstaging to M1 disease.^21^

## Current best practice in imaging to assess response

CT is routinely performed, and ^18^F-FDG PET/CT in some centres, after neoadjuvant therapy to assess response, although in current practice, this is simply to ensure that disease has not progressed and become unresectable (either due to advanced T-stage or development of metastases). Up to 10% of patients with potentially resectable oesophageal cancer develop metastases whilst on neoadjuvant chemotherapy.^22,23^ However, complete pathological response is reported in 32–52% depending on the criteria used^24–26^ and a management aspiration is that some of these patients may be candidates for surveillance programmes rather than oesophagectomy in future.

Contrast-enhanced CT has low sensitivity for residual disease, so cannot adequately assess for treatment response.^27^ EUS is also of little value in restaging after neoadjuvant therapy because post-treatment inflammation and fibrosis can be indistinguishable from residual tumour.^28^ A further challenge is that nodal response can be discordant with the primary tumour (in approximately 5%), and prognosis may be improved in cases with improved nodal disease, even in the absence of response in the primary tumour.^29^ Conventional morphological imaging may struggle to assess this, as even morphologically normal lymph nodes can contain metastasis. Foley et al reviewed resection specimens in 15 patients pre-operatively staged as N0 but with nodal metastases found on pathological assessment. In 50 nodal metastases, 22% were 2 mm or less, and 82% were 6 mm or less, implying that novel methods that augment morphological assessment are required.^30^

## Current best practice in follow-up & suspected recurrence

Following treatment, the final challenge for imaging is to monitor for disease relapse. Despite multimodality therapy, there are high rates of post-treatment relapse, reported at 45–53% within 2 years of surgery.^31,32^ Anastomotic recurrence following surgery occurs in 7–12%, seen as nodular or concentric thickening in the region of the anastomosis on CT.^31,33^ In one study of recurrence following oesophagectomy, 50% of the 435 recurrences were detected as a result of symptoms, and 45% were as a result of routine post-treatment CT studies.^34^ The role of cross-sectional imaging in the surveillance of patients post-oesophagectomy is contentious and further research is required to standardise practice.

^18^F-FDG PET/CT has the highest sensitivity for recurrent disease at 89–100%, although specificity is much lower (55–94%).^33^ Local inflammation in the oesophagus can cause false positive FDG-uptake and should be confirmed with endoscopy.^35^ The radiation doses associated with PET/CT mean it is currently used as a problem-solving tool in the setting of indeterminate findings on CT, rather than as part of a routine surveillance programme.

## State-of-the-art imaging in staging

The combined limitations of CT, ^18^F-FDG PET/CT and EUS mean that new imaging technologies are needed to improve the delineation of disease extent, the detection of lymph node metastases, and the assessment of treatment response. There is an opportunity for state-of-the-art imaging techniques to address these gaps, ensuring that patients are stratified to the most appropriate treatment.

### MRI

**Figure 3.**
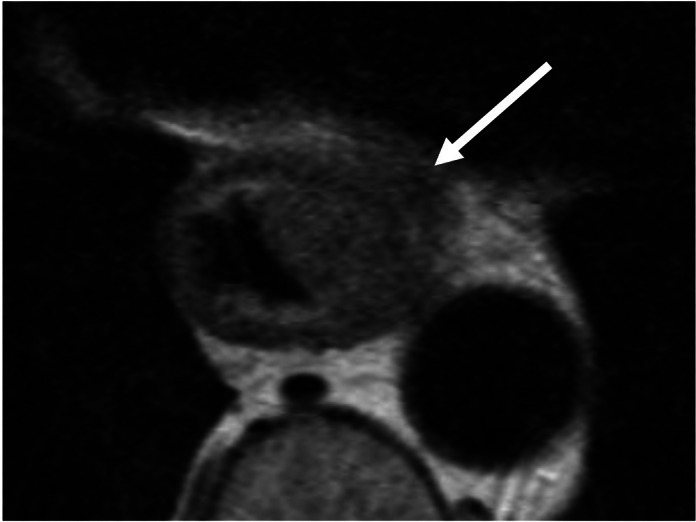
Small field-of-view axial *T*_2_ weighted MRI. There is a primary tumour centred on the left-side of the oesophagus extending through the muscularis into the perioesophageal fat (arrow). The normal intact layers of the oesophageal wall are seen on the contralateral side (Image courtesy of Dr A M Riddell, Royal Marsden Hospital).

MRI has excellent soft tissue contrast and can identify the normal layers of the oesophageal wall (Figure 3), with potential to improve stratification of patients towards endoscopic resection, upfront surgery or neoadjuvant therapy. In seminal work, Riddell et al showed *T*_2_ weighted images from 1.5 T MRI were comparable to EUS in differentiating T2 from T3, but overstaged T1 tumours. Overall, 83% (*n* = 28/37) were staged correctly against histology; 16% were overstaged and 8% understaged.^36^ *Ex-vivo* studies at ultra-high field strength (4.7 T and 7 T) have shown up to 100% accuracy for T- and N-stage, although application to clinical practice has not been tested.^37^ MRI with diffusion-weighted sequences (DWI) has the potential to improve staging accuracy and assessment of tumour length.^38^ A recent systematic review identified 984 patients in 19 studies and found that MRI (without any restriction on the sequences used) had a sensitivity of 67–91% and specificity of 91–92% for differentiating T2 tumours or less from T3 or above, compared to 85 and 75% for CT, and 68–100% and 75–100% for EUS.^38–40^

Notably, oesophageal MRI is challenged by organ peristalsis, cardiac and respiratory motion, aortic blood flow and pulsation, and artefact from medical devices. Additionally, due to its central position within the thorax, the oesophagus is distant from MR coils which lowers the signal-to-noise ratio.^41^ Use of 3 T MRI systems can increase the signal-to-noise ratio but at the cost of increased susceptibility artefact. Cardiac and respiratory gating and endoluminal or surface coils can improve image quality.^36,42^

In terms of differentiating node negative from node positive disease, MRI has a reported sensitivity of 59–100% and specificity 57–92%, dependent on the size threshold used to define a metastatic lymph node,^39^ compared to 83 and 75% for CT, 46 and 91% for PET/CT, and 100 and 36% for EUS.^38,39,43^ In addition to size criteria, alternative imaging features have been proposed to differentiate malignant *vs* normal nodes. Alper et al used quantitative assessment of STIR (short tau inversion recovery) MRI to investigate nodal involvement in 35 patients, specifically looking at the signal intensity ratio between each lymph node and the normal oesophageal wall. Compared to histology, 152 of 482 nodes were detected by MRI, with the signal intensity ratio significantly higher in pathological nodes.^44^ MRI enhanced with superparamagnetic iron oxide nanoparticle (SPIO) contrast has the potential to differentiate normal or reactive from metastatic lymph nodes because normal nodes contain substantial phagocytosed SPIO, which appears low signal due to magnetic susceptibility and T2* shortening, whereas malignant nodes are intermediate signal.^45^ In oesophageal cancer, one study of nine patients showed this to be a feasible technique,^46^ although concerns around safety in clinical practice have been raised.^47^

### Whole-body MRI

Following the success of the STREAMLINE-C trial in colorectal cancer,^48^ the role of whole body-MRI in oesophago-gastric cancer staging has been raised. Whole-body MRI might allow a more streamlined and cost-effective staging pathway, with local and distant staging of disease having equivalent accuracy to current investigations in a single radiological examination. However, high-quality studies in oesophago-gastric cancer are lacking. In one small study, whole-body MRI had a reported equivalent accuracy to PET/CT for N-stage and excluding metastatic disease (*n* = 49 patients, using surgical specimens or EUS for N-stage, and metastases being detected in only two patients and with both modalities).^43^ This has not yet been tested within a rigorous clinical trial. In systematic review, albeit in gastric cancer, whole-body MRI was comparable to CT in detection of peritoneal disease, which is a common metastatic site from oesophageal cancer.^49,50^ The addition of DWI in a whole-body MRI protocol improves sensitivity over morphological images alone (90% *vs*  73% in assessment of 255 peritoneal deposits in 34 patients, verified at laparoscopy and with histology, but again not in oesophageal cancer).^51^

### Integrated PET/MRI

**Figure 4.**
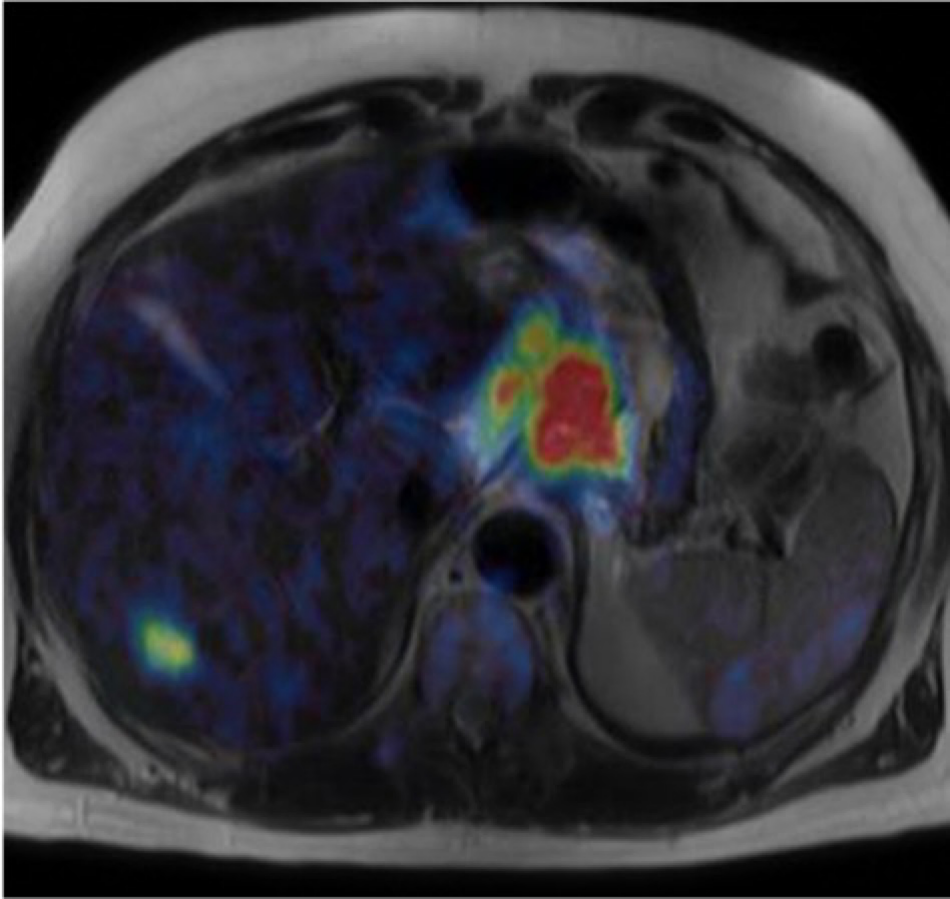
Axial fused ^18^F-FDG PET/MRI image showing FDG uptake in the primary tumour, locoregional nodes and liver metastasis in a patient with Stage IV oesophago-gastric cancer (Images courtesy of Professors G Cook and V Goh, St. Thomas’ Hospital). FDG, fluorodeoxyglucose.

^18^F-FDG PET/MRI combines the benefits of both ^18^F-FDG PET/CT and MRI, i.e. optimising locoregional and distant staging in one examination (Figure 4). Lee et al found a diagnostic accuracy of 67% for T-stage compared to 87% for EUS in a prospective study of 19 patients with pathological correlation. ^18^F-FDG PET/MRI N-stage accuracy was 87% compared to 67% for EUS, and 50% for CT.^52^ Good agreement between ^18^F-FDG PET/MRI and PET/CT for N- and M-stage has been reported.^53^ Sharkey et al compared TNM stage between tumour board consensus (from all available diagnostic tests, excluding PET/MRI), ^18^F-FDG PET/CT alone, and ^18^F-FDG PET/MRI alone. In this prospective study of 22 patients, 10 with metastatic disease, additional metastases were found on ^18^F-FDG PET/MRI in 30% of cases compared to PET/CT (two peritoneal and one liver), which has potential clinical relevance when detection of a single metastatic site can change management between curative and palliative therapy.^54^ ^18^F-FDG PET/MRI can be time-saving compared to acquiring the images separately,^55^ however, this strategy assumes that patients will require a PET examination during their staging. Potential imaging biomarkers from ^18^F-FDG PET/CT (glucose metabolism) and MRI (perfusion phenotype from dynamic contrast-enhanced (DCE) MRI or cellularity from DWI) are discussed below. Acquisition of these modalities contemporaneously could be advantageous.

## State-of-the-art imaging in treatment planning

**Figure 5.**
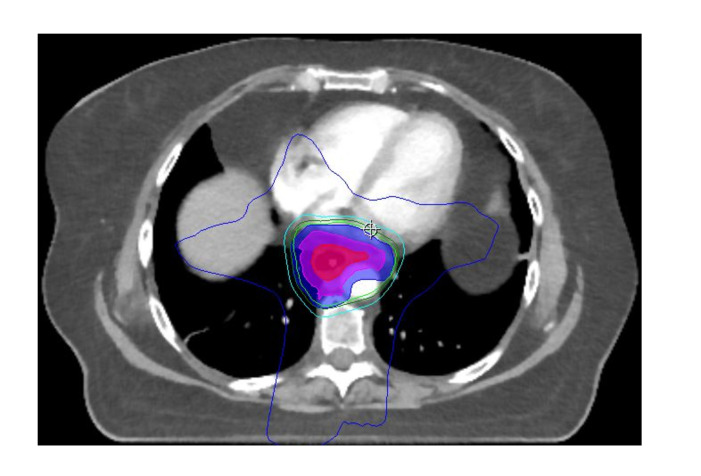
CT-based radiotherapy planning image of a distal oesophageal tumour demonstrating the GTV (red), CTV (pink) and PTV (blue) with several isodose lines contoured by clinical oncologists during target volume delineation (Images courtesy of Dr Owen Nicholas, South West Wales Cancer Centre). CTV, clinical target volume; GTV, gross tumour volume; PTV, planning target volume.

In oesophago-gastric cancer, treatment decisions are based on a number of factors including disease stage, patient fitness and preferences, age and pathology. Neoadjuvant therapy prior to resection is the standard of care for patients with Stage II to III disease, offering a 5.1% absolute survival advantage at 2 years *vs* surgery alone in adenocarcinomas, highlighting the importance of accurate staging.^56^ However, only a minority of patients (14.8%) demonstrate a good response to treatment, defined as a tumour regression grade (TRG) of 1 or 2.^57^ Further, disease length is an important consideration when planning operation type or suitability for radiotherapy, the latter being dependent on a maximum field length of approximately 12 cm.^58^ CT is traditionally used to define the tumour and organs at risk during radiotherapy planning, however, ^18^F-FDG PET/CT has now been incorporated into the planning process and uses the metabolic activity of the primary tumour and nodes to adjust the irradiated volumes.^59^ When performed, EUS provides measurements defining the location of disease and important anatomical landmarks such as the aortic arch, carina and diaphragm which can be used to augment the radiotherapy plan (Figure 5). There is an opportunity for state-of-the-art imaging and biomarkers to improve patient selection for individualised treatment, enabling prediction of the likelihood of response, expediting patients for surgery who are unlikely to respond, and improved definition of disease.

## State-of-the-art imaging in predicting response and outcome

### Diffusion-weighted MRI

Apparent diffusion coefficient (ADC) values are lower in areas of increased cellularity such as tumour. There have been conflicting reports regarding the predictive value of pre-treatment ADC values. Aoyagi et al. (*n* = 80; SCC undergoing chemoradiotherapy) reported that higher pre-treatment ADC values (1.1 × 10^−3^ mm^2^/s and greater) were associated with increased likelihood of response assessed by RECIST (hazard ratio 23.4 (6.1–89.6)) and increased survival (42% 1 year survival versus 18%) in advanced SCC,^60^ whereas De Cobelli et al. (*n* = 32 oesophageal and gastric cancers; 81% adenocarcinoma; undergoing either chemoradiotherapy or chemotherapy, respectively) found responders to neoadjuvant therapy assessed by Mandard TRG had significantly lower pre-treatment ADC values (mean ADC in responders (TRG 1–3) was 1.32 ± 0.33 x 10^−3^mm^2^/s *vs* 1.63 ± 0.41 x 10^−3^mm^2^/s in non-responders. *p* = 0.002).^61^

### Perfusion imaging

**Figure 6.**
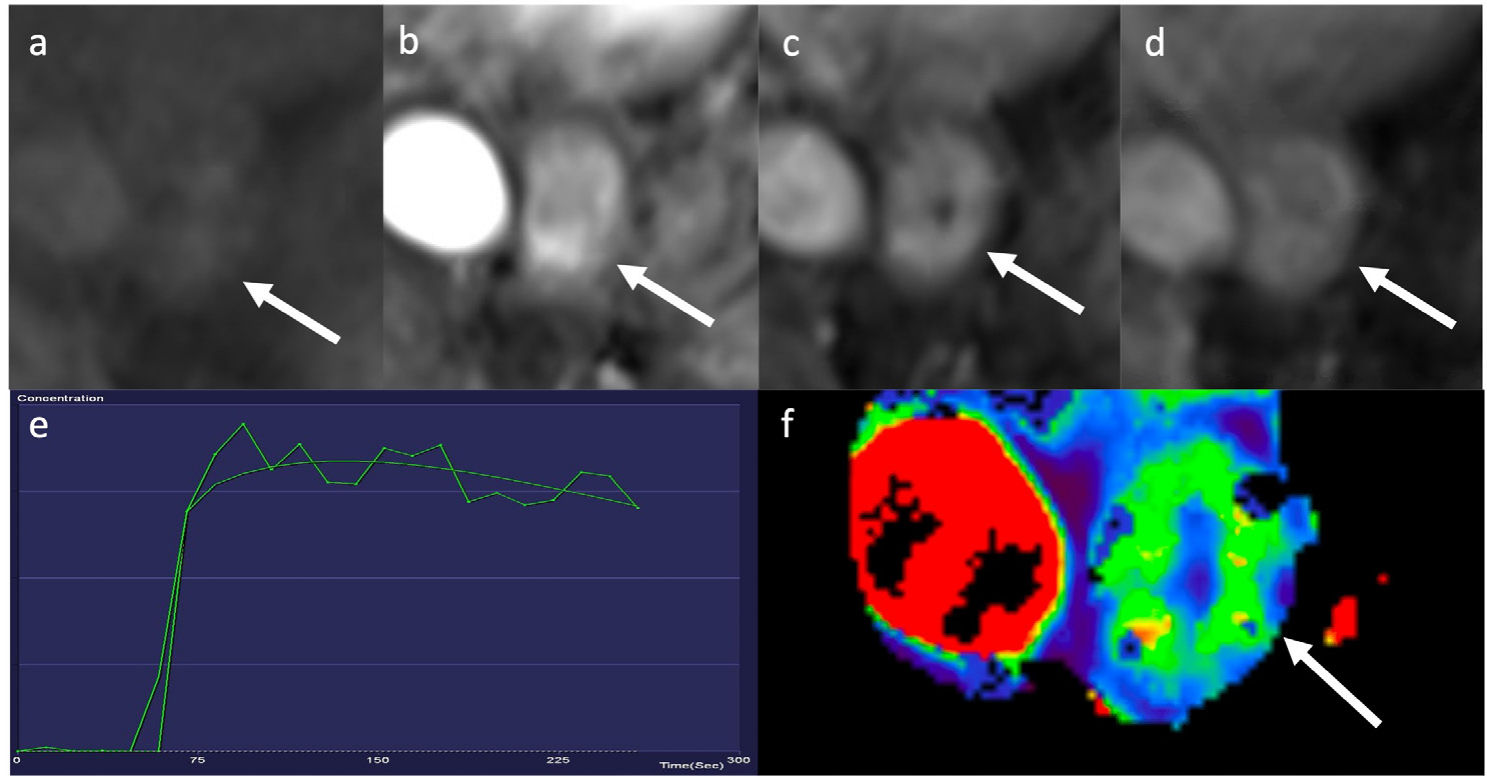
Axial fat-saturated *T*_1_ weighted MRI performed before (a), 30 s (b), 70 s (c) and 120 s (d) after contrast administration in a patient with a distal oesophageal tumour (arrows). Contrast-enhancement curve showing a type-3 washout curve (e). Focussed K^trans^ map showing mean K^trans^ value of 0.39 min^−1^ within the oesophageal tumour (arrow) (f). (Images courtesy of Professor V Goh, St. Thomas’ Hospital).

Perfusion parameters from DCE-MRI or perfusion CT are a further potential avenue to predict response. The transfer constant (K^trans^) is the rate of leakage of contrast into the interstitium and is related to blood flow and tissue permeability (Figure 6). In many studies, K^trans^ is the DCE-MRI parameter which is most predictive of response to treatment. Higher pre-treatment K^trans^ values have been associated with better response to neoadjuvant and palliative chemoradiotherapy (Sun et al *n* = 59 SCC and Lei et al *n* = 25 SCC).^62,63^ Heethuis et al. (*n* = 25, 84% adenocarcinoma) found significantly different pre-treatment MRI perfusion parameters (25% percentile of iAUC) in good pathological responders.^64^ Using perfusion CT, several authors have found increased blood flow associated with an increased likelihood of response. Hayano et al (*n* = 31 SCC) reported mean blood flow 90.2 ml/100 g/min in responders, defined by >50% tumour volume decrease, *vs* 35.9 ml/100 g/min in non-responders (*p* = 0.0004).^65^ Zhao et al. (*n* = 27 SCC) found responders, assessed by RECIST, had mean blood flow 42.1 *vs* 27.5 ml/100 g/min (*p* = 0.007) for non-responders.^65,66^ It is hypothesised that better perfused tumours may receive higher doses of chemotherapeutic agents which has direct relevance for treatment selection.

### PET/CT

Published data on the value of ^18^F-FDG PET/CT parameters in prediction of response or survival are heterogeneous and conflicting. Authors have reported that increased maximum standardised uptake value (SUV_max_) is associated with increased likelihood of response^67^ whilst others found the opposite^68^ or no relationship at all.^69^

In addition to ^18^F-FDG, there are opportunities for use of novel PET tracers in oesophago-gastric cancer. Human epidermal growth receptor 2 (HER2-neu) is overexpressed in many oesophageal adenocarcinomas. Monoclonal antibodies targeting this receptor have been developed as immunotherapy-based treatments, *e.g.* Trastuzumab, which has been shown to improve overall survival in the metastatic setting.^70^ However, heterogeneity of metastatic sites is not a unique problem to oesophago-gastric cancer and can lead to treatment failure. In a study of 33 patients undergoing both 89Zr-Trastuzumab PET/CT and ^18^F-FDG PET/CT, an average of 5.5 lesions per patients were identified using 89Zr-Trastuzumab PET/CT compared to 8 using ^18^F-FDG PET/CT.^71^ This shows the potential of novel imaging tracers to promote and select patients for individualised treatment plans. Further, the possibility of theranostic treatment options using 177Lu-Trastuzumab are being researched for HER2 positive breast cancer,^72^ which may translate to oesophago-gastric cancer. Potential other molecular targets include vascular endothelial growth factor, epidermal growth factor receptor^73^ and hypoxia imaging.^74^

### State-of-the-art imaging in assessing response

Conventional imaging cannot adequately predict treatment response with sufficient accuracy to change current clinical pathways. There are opportunities for state-of-the-art imaging techniques to meet this challenge and improve treatment response assessment. A further role of imaging could be to predict response at an earlier time point during neoadjuvant therapy, to allow changes to the treatment regimen, or an alternative approach.

### Diffusion-weighted MRI

**Figure 7.**
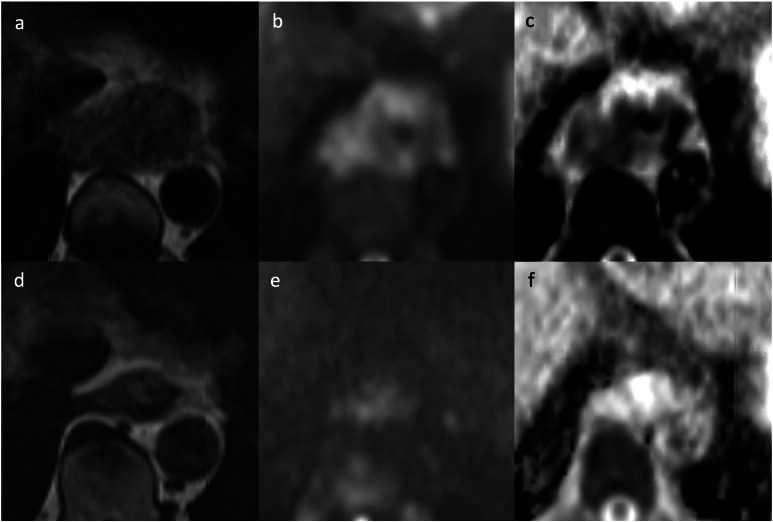
Axial *T*_2_ weighted MRI, B900 diffusion-weighted MRI, and apparent diffusion coefficient map before (a, b, c) and after (d, e, f) neoadjuvant chemoradiotherapy. The tumour is at the gastro-oesophageal junction, above a moderate-sized hiatus hernia. The tumour decreased in bulk, and mean tumour apparent diffusion coefficient increased from 800 × 10^−3^ mm^2^/s to 1850 × 10^−3^ mm^2^/s (Images courtesy of Professor V Goh, St. Thomas’ Hospital).

MRI does not involve ionising radiation, therefore can be used repeatedly throughout the staging and treatment pathway. Changes in tumour ADC values during neoadjuvant treatment have been reported as a potential method of predicting response with early increases in ADC associated with favourable response (Figure 7).^75,76^ van Rossum et al (*n* = 20; 75% adenocarcinoma) reported that less than 29% ADC increase had 100% sensitivity for residual tumour at the end of neoadjuvant therapy.^76^ Heethuis et al (*n* = 45; 84% adenocarcinoma) reported that ADC values increased during treatment in both good and poor responder groups, although the change was more marked in good responders (23.5 ± 20.5% and 9.8 ± 11.7%, respectively, *p* = 0.035).^77^ Borggreve et al performed DWI before and weekly during neoadjuvant chemoradiotherapy to determine the optimum time to predict complete pathological response (TRG 1). Seven of 24 patients had complete response. The percentage increase in ADC from baseline to the 2-week scan was most predictive of subsequent complete response (mean increase 36% in complete response *vs* 16% in non-complete response), with a c-index of 0.87, which increased to 0.97 after exclusion of small tumours.^78^ Visual assessment on post-treatment DWI had good sensitivity for detection of residual local disease, but low specificity (42–50%).^79^ Some authors have found an inverse relationship between ADC values and TRG, with higher post-treatment ADC values associated with better response (de Cobelli et al *n* = 32 oesophageal and gastric cancers; and more recently Giganti et al *n* = 18 oesophageal, including some of the same patients as the study by de Cobelli et al).^38,61^

### Dual energy CT

Dual energy CT can provide quantitative information about iodine contrast uptake. A full review of different dual energy CT methodologies is described by McCollough et al^80^ but in brief it utilises varying X-ray energy spectra to simultaneously (either with two separate X-ray sources or with rapid kV switching) or sequentially acquire two sets of image data at the same anatomical location. This can provide more information on the constituent materials present within a voxel as the images are combined with spectral characterisation.^81^ In a single centre study of 45 patients, iodine concentrations after chemoradiotherapy were significantly lower in patients that responded *vs* non-responders (assessed by RECIST v 1.1).^82^

### Perfusion imaging

Decreased blood flow^65,83^ and increased mean transit time^84^ have been associated with improved response to chemoradiotherapy and increased survival. In a single centre study of 40 SCC patients following neoadjuvant therapy, Djuric-Stefanovic et al found increased blood flow and increased blood volume were associated with higher Mandard TRG scores (*i.e.* worse response). They found blood flow <30 ml/min/100g corresponded with complete pathological response.^85^

Using DCE-MRI, Heethuis et al (*n* = 45, 84% adenocarcinoma) found the change in iAUC (the initial area under the Gadolinium concentration curve, reflecting contrast inflow and vascular leakage) between pre-treatment MRI and MRI performed after one cycle of neoadjuvant therapy could discriminate between TRG 1–2 and 3–5 (10.6±17.6% increase in iAUC in complete responders *vs* 45.2 ± 41.5% increase in non-complete responders. *p* = 0.028).^77^ In oesophageal SCC (*n* = 59) treated with chemoradiotherapy and assessed with DCE-MRI before and after treatment, the absolute post-treatment K^trans^ (the transfer constant, reflecting the rate of leakage of Gadolinium from vessels to the interstitium) values were lower in complete responders (by RECIST v 1.1) *vs* the remainder, and the decreases in K^trans^ and K_ep_ (the rate constant, reflecting the rate of return of contrast back from the interstitium) values were significantly greater in complete responders.^63^ Other studies (*n* = 25 SCC) found significantly lower K^trans^ and higher V_e_ (the relative volume of extracellular extravascular space available to accumulate Gadolinium) in complete responders *vs* partial response when assessed by RECIST v 1.1.^62^ Conversely, Heethuis et al did not find post-treatment iAUC was able to differentiate complete pathological responders *vs* non-complete responders.^77^ A combination of DCE-MRI (change in K^trans^ between per- and pre-treatment) and DW-MRI (change in ADC between post- and pre-treatment) has yielded better performance than either individually (c-index 0.89 *vs* 0.79 and 0.75, respectively).^77^

### PET/CT

Published data on the value of ^18^F-FDG PET/CT parameters for predicting response or survival are heterogeneous. PET/CT performed during treatment has been shown to predict response as metabolic changes can precede pathological response. In a 2017 meta-analysis including 13 studies and 697 patients, 8 studies found some predictive value in performing PET/CT during neo-adjuvant chemoradiotherapy, whereas 5 did not. Pooled sensitivity for complete pathological response was 63–100% and specificity 50–76%.^86^ One study used early PET/CT during neoadjuvant chemotherapy to identify metabolic non-responders. Patients with greater than 35% decrease in SUV_max_ continued with the standard pathway, but those with less than 35% decrease were considered non-responders and proceeded straight to surgery, showing the potential of PET/CT to alter the neoadjuvant treatment pathway. Of 54 patients classified as non-responders on early PET/CT, none had a major histological response (<10% viable tumour remaining) at the time of resection.^87^ Metabolic responders had better progression-free survival (29.7 *vs* 14.1 months). To determine the effect of early PET/CT for treatment change, it would be necessary to randomise metabolic non-responders to either continued standard care or early surgery, which is currently lacking and may not be feasible.

^18^F-FDG PET/CT performed after neoadjuvant therapy can be challenging to interpret as radiation-induced oesophagitis or ulceration is common and also has FDG uptake, although this will generally resolve within 6–12 weeks of radiotherapy.^88^ Despite PET/CT having the highest sensitivity for identifying distant metastases, up to 5% of patients will have false-positive sites of uptake requiring further imaging or biopsy.^89^ Equally, a lack of FDG uptake at the primary site cannot distinguish between microscopic residual disease and complete pathological response.^90^ In one study of 135 patients with negative biopsies after neoadjuvant treatment, 85 had residual tumour at oesophagectomy.^91^ As part of the prospective preSANO trial, 129 patients underwent ^18^F-FDG PET/CT after neoadjuvant chemoradiotherapy and prior to surgery. They were able to identify complete response (TRG 1) with 80% sensitivity and 37% specificity. 15% of patients with a poor response (TRG 3 or 4) had a complete metabolic response on PET/CT.^23^ A study by Cerfolio et al used an SUV_max_ cut-off of 3.25 and found this predicted complete response with 67% sensitivity and specificity.^92^ Further, FDG PET/CT was significantly better at predicting complete response than EUS and CT (89% vs 67 and 71%, respectively).^92^

Novel PET tracers could play a potential role in predicting response by investigating changes in uptake values early during treatment. ^18^F-Fluorothymidine is a PET tracer that acts as a marker of cellular proliferation and is thought to discriminate between tumour and inflammation. Chen et al (*n* = 34 SCC) found a decrease in ^18^F-Fluorothymidine uptake 4 weeks after starting neoadjuvant chemoradiotherapy for SCC was associated with improved locoregional control and better 2 year progression-free survival, whereas ^18^F-FDG uptake was not predictive.^93^ In a pilot study of 26 patients undergoing both ^18^F-FDG PET/CT and 11C-thiothymidine before and after neoadjuvant treatment, lower post-treatment 11C-thiothymidine SUV_max_ and a greater percentage decrease in 11C-thiothymidine SUV_max_ were associated with pathological response. Similarly, the percentage change in ^18^F-FDG SUV_max_ was also associated with pathological response, however absolute ^18^F-FDG SUV_max_ was not.^94^

### State-of-the-art imaging of recurrent disease

Only small studies have compared MRI *vs* CT in the setting of recurrent disease. In 23 patients with recurrent disease following oesophagectomy, CT and MRI performed equally well at identifying intraluminal local recurrence, liver metastases, and malignant pleural and pericardial effusions. MRI identified more bone metastases and was superior in identifying malignant oesophageal wall thickening but performed worse in assessment of lung metastases.^95^ Shuto et al investigated DWI in assessment of nodal relapse (*n* = 47 suspected nodes identified on CT, with histology as reference standard), finding restricted diffusion assessed on ADC map had diagnostic accuracy of 81% compared to 87% from ^18^F-FDG PET/CT.^96^

## Conclusions

State-of-the-art imaging techniques have the potential to transform the diagnostic, staging and treatment pathway for patients with oesophago-gastric cancer. Whole body MRI, PET/MRI, and novel PET tracers have shown promise in early research studies of oesophago-gastric cancer and may allow more precise delineation of disease extent and prediction of treatment response thus optimising treatment decisions and patient outcomes.
